# Diagnostic Accuracy of GeneXpert MTB/RIF Assay in Comparison to Conventional Drug Susceptibility Testing Method for the Diagnosis of Multidrug-Resistant Tuberculosis

**DOI:** 10.1371/journal.pone.0169798

**Published:** 2017-01-12

**Authors:** Pratikshya Pandey, Narayan Dutt Pant, Komal Raj Rijal, Bhawana Shrestha, Sirita Kattel, Megha Raj Banjara, Bhagwan Maharjan, Rajendra KC

**Affiliations:** 1 Department of Microbiology, Mark Formulations Pvt. Ltd., Kathmandu, Nepal; 2 Department of Microbiology, Grande International Hospital, Dhapasi, Kathmandu, Nepal; 3 Central Department of Microbiology, Tribhuvan University, Kathmandu, Nepal; 4 German Nepal Tuberculosis Project- National Reference Laboratory, Kathmandu, Nepal; 5 Department of Biochemistry, Modern Technical College, Lalitpur, Nepal; St Petersburg Pasteur Institute, RUSSIAN FEDERATION

## Abstract

Xpert MTB/RIF assay is regarded as a great achievement of modern medicine for the rapid diagnosis of multidrug-resistant tuberculosis (MDR-TB). The main purpose of this study was to determine the performance of Xpert MTB/RIF assay compared to conventional drug susceptibility testing (DST) method for the diagnosis of MDR-TB. A comparative cross sectional study was carried out at German-Nepal Tuberculosis Project, Kathmandu, Nepal, from April 2014 to September 2014. A total of 88 culture positive clinical samples (83 pulmonary and 5 extra-pulmonary) received during the study period were analyzed for detection of multidrug-resistant tuberculosis by both GeneXpert MTB/RIF assay and conventional DST method. McNemar chi square test was used to compare the performance of Xpert with that of DST method. A p-value of less than 0.05 was considered as statistically significant. Of total 88 culture positive samples, one was reported as invalid while 2 were found to contain nontuberculous Mycobacteria (NTM). Among remaining 85 *Mycobacterium tuberculosis* culture positive samples, 69 were found to be MDR-TB positive by both methods. The overall sensitivity, specificity, positive predictive value (PPV) and negative predictive value (NPV) of GeneXpert MTB/RIF assay were found to be 98.6%, 100%, 100% and 93.8% respectively. Statistically, there was no significant difference between the diagnostic performance of Xpert and conventional DST method for detection of MDR-TB. GeneXpert MTB/RIF assay was found to be highly sensitive, specific and comparable to gold standard conventional DST method for the diagnosis of MDR-TB.

## Introduction

Tuberculosis (TB) presents as a serious public health problem, globally with an estimated 10.4 million new TB cases in 2015 [1]. It is a leading cause of deaths from infectious diseases (with worldwide estimated 1.8 million deaths in 2015), large numbers of deaths mainly occurring in low and middle income countries [1]. In 2015, 480,000 new MDR-TB cases were estimated to occur worldwide [1].

Worldwide, large percentage of cases of MDR-TB remain undiagnosed. Further, there is an increasing trend of prevalence of MDR-TB in Nepal. According to the recent drug resistance surveillance in Nepal, the prevalence of MDR-TB was 2.6% in case of newly diagnosed tuberculosis cases and17.6% among previously treated cases [2].

The conventional *Mycobacterium tuberculosis* (MTB) drug susceptibility testing is a gold standard technique but requires long period of time (8 weeks) to give definitive report [3]. Further, it requires more sophisticated and higher bio-safety level laboratory along with the well trained staffs [3]. Therefore, the diagnosis of MDR-TB by using the conventional drug susceptibility testing is not possible in resource limited countries like Nepal [3]. In addition, due to the very slow turnaround time of conventional technique the chances for early diagnosis of MDR-TB and proper patient management is very low even in the settings where there is availability of resources for performing the gold standard technique [3]. To address these issues, Xpert MTB/RIF assay was developed and it was endorsed by world health organization (WHO) in December 2010 as an accurate, feasible, rapid, affordable, and near-point-of-care TB diagnostic test for use in resource-limited settings [4]. Many studies on DNA sequencing have reported that more than 95% of rifampicin (RIF) resistant MTB isolates have mutation within the 81-bp hot spot region of the *rpoB* gene [5, 6, 7]. The Xpert assay works by detecting MTB and RIF resistance by polymerase chain reaction (PCR) based amplification of the 81-bp *rpoB* gene segment and probing for the mutations that are related to RIF resistance. The assay is automated and completes within 2 hrs [8, 9]. After the redesign of probe B in December 2011 [10], studies have assessed the performance of Xpert in detection of MTB and MDR-TB. We also aimed to evaluate the diagnostic accuracy of Xpert MTB/RIF assay in comparison to conventional DST for diagnosis of MDR-TB in our setting.

## Materials and Methods

### Data availability

All relevant data are within the paper.

### Ethics statement

The present study was approved by ethical committee of Nepal Health Research Council (NHRC). All the patients or patient’s guardians were informed of the potential use of patient’s clinical specimens for research and written informed consent was obtained.

### Study design

A comparative cross sectional study was conducted among patients attending German Nepal Tuberculosis Project (GENETUP) laboratory, Kathmandu, Nepal, from April 2014 to September 2014. The clinical samples from all the 88 culture positive TB patients received during the study period from all over Nepal were used in our study. Out of total 88 culture positive clinical specimens(83 pulmonary and 5 extra pulmonary), 2 were reported to contain NTM and 1 as invalid since no growth was observed in control of its DST. So, the remaining 85 MTB culture positive samples were processed for determination of rifampicin resistance by conventional DST and GeneXpert MTB/RIF assay and the results were compared. Out of total 85 MTB culture positive clinical specimens, 37 were from newly diagnosed TB cases (TB cases in which TB was diagnosed for the first time) and 48 were from previously treated TB cases (TB cases in which TB was diagnosed and treated previously). Performance of GeneXpert MTB/RIF assay was assessed by computing sensitivity, specificity, PPV and NPV of the test with respect to the gold standard conventional DST method.

### Detection of MDR-TB

Diagnosis of MDR-TB was performed by conventional DST method [11] and GeneXpert MTB/RIF assay.

### GeneXpert MTB/RIF assay

To 0.5ml of sample, Xpert sample reagent (SR) was added in the ratio 1:3 for decontaminated sample and 1:2 for direct sample and was shaked vigorously twice during 15 minutes incubation at room temperature. Two ml of the mixture was transferred to Xpert test cartridge and the cartridge was then loaded into Xpert device. Finally, the results were interpreted by the GeneXpert DX system from measured fluorescent signals and displayed automatically after 90 mins.

### Statistical analysis

For the analysis of the data obtained, SPSS 16.0 was used. McNemar Chi square test was used to find the significant differences between two methods at 95% confidence interval. A p-value less than 0.05 was considered statistically significant.

## Results

### Summary of anti-tubercular drug susceptibility test

Out of 88 culture positive clinical specimens, 55 were from male, 83 were from pulmonary TB cases and 37 were from newly diagnosed TB cases. The age of culture positive TB patients ranged from 13–82 years with the highest percentage in productive age group i.e. 15–45 years (71.6%) (p<0.05). Out of 87 *Mycobacterium* spp. isolated from culture positive clinical specimens (excluding the invalid sample), only 16% (n = 14) were sensitive to all 4 drugs (isoniazid, rifampicin, streptomycin, ethambutol) tested and 64.4% (n = 56) were found to be resistant to all 4 drugs. Almost, 82.8% (72/87) of the *Mycobacterium* spp. isolated were found to be resistant to isoniazid followed by rifampicin (81.6%) (Table 1).

**Table 1:** Summary of anti-tubercular drug susceptibility test.

	Total	Gender		Sample Category		Patients Category	
		Male	Female	Pulmonary	Extra-pulmonary	New	Retreatment
Total tested	87	55	32	82	5	37	50
Sensitive to all 4 drugs	14(16.1%)	10(18.2%)	4(12.5%)	12(14.6%)	2(40%)	5(13.5%)	9(18%)
Resistant to all 4 drugs	56(64.4%)	35(63.6%)	21(65.6%)	53(64.6%)	3(60%)	27(73%)	29(58%)
Resistant to at least 1 drug	73(83.9%)	45(81.8%)	28(87.5%)	70(85.4%)	3(60%)	32(86.5%)	41(82%)
Resistant to Rifampicin	71(81.6%)	43(78.2%)	28(87.5%)	68(82.9%)	3(60%)	32(86.5%)	39(78%)
Resistant to Isoniazid	72(82.8%)	44(80%)	28(87.5%)	69(84.1%)	3(60%)	32(86.5%)	40(80%)
Resistant to Ethambutol	59(67.8%)	37(67.3%)	22(68.8%)	56(68.3%)	3(60%)	28(75.7%)	31(62%)
Resistant to Streptomycin	66(75.9%)	41(74.5%)	25(78.1%)	63(76.8%)	3(60%)	30(81.1%)	36(72%)

### Performance of GeneXpert as compared to gold standard conventional drug susceptibility test

Diagnostic efficacy of GeneXpert was assessed in 85 MTB culture positive cases with the overall sensitivity, specificity, PPV and NPV at 95% confidence interval of 98.6% (92.3–99.8), 100% (78–100), 100% (94.7–100) and 93.8% (69.7–98.9) respectively. Only 1/85 and 2/85 cases reported as RIF sensitive by GeneXpert assay were found to be resistant to RIF and isoniazid (INH) respectively by conventional DST. Statistically, there was no significant difference between Xpert MTB/RIF assay and conventional DST method for detection of MDR-TB, (p>0.05) (Table 2).

**Table 2:** Performance of GeneXpert as compared to gold standard conventional drug susceptibility test.

GeneXpert MTB/RIF Assay	Drug Susceptibility Test (rifampicin and isoniazid)			Sensitivity (95% CI)	Specificity (95% CI)	PPV (95% CI)	NPV (95% CI)	P-value
	Resistant	Sensitive	Total	98.6% (92.3–99.8)	100% (78–100)	100% (94.7–100)	93.8% (69.7–98.9)	1.00
Resistant	69(81.2%)	0	69(81.2%)					
Sensitive	1(1.2%)	15(17.6%)	16(18.8%)					
Total	70(82.4%)	15(17.6%)	85					

## Discussion

In a Cochrane review [12], Xpert MTB/RIF pooled sensitivity for rifampicin resistance detection was 95% (95% CI, 90% to 97%; 17 studies) which was found to be lower than in our study i.e. 98.6% (95% CI, 92.3–99.8) and pooled specificity was 98% (95% CI, 97% to 99%; 24 studies) which was again lower than our study i.e. 100% (95% CI, 78–100). One case found to be positive for resistance to RIF phenotypically (detected by conventional DST method), was found to be negative for resistance to RIF genotypically by Xpert. The reason for this discrepancy might be due to mutation in other sites rather than hot spots, which needed to be solved by further sequencing the rpoB gene. Further, in our study no false positive cases were detected. This is the strength in favor of using Xpert for the detection of RIF resistance.

Furthermore, Xpert has very short turnaround time (<2 hrs) with low biohazard risk [13]. Rifampicin resistance can be detected in less than one day with Xpert MTB/RIF assay while it takes an average of 75 days for diagnosis of MDR-TB with the help of conventional DST [14]. And of much concern about in this study is 86.5% (32/37) of the new and 79.2% (38/48) of the retreatment TB patients had MDR-TB, which indicates the high level of resistance even when the patients were first infected and this high rate of MDR-TB in new cases might be due to sample selection bias, sample being taken from the TB reference center where culture positive cases from various geographical regions are referred. Further, the other reason for this may be due to patients being reluctant in providing information about their previous exposure and treatment failure.

### Limitations of the study

Inability to include large numbers of different samples is a major limitation of the study. If large numbers of the different samples could have been used, it would have been easy for performing subgroup analysis comparing the results of different subgroups. In addition, the use of large number of samples from different geographical regions would have generated more significant data. However, in our study samples were taken from the TB reference center where culture positive cases from various geographical regions are referred.

## Conclusions

The sensitivity and specificity of GeneXpert MTB/RIF assay for the diagnosis of MDR-TB was found to be comparable to gold standard, conventional DST. Further, due to short turnaround time, it is highly significant to use GeneXpert MTB/RIF assay for diagnosis of MDR-TB in country with high prevalence of MDR-TB. It will be helpful to TB control program by helping in early case finding in country like Nepal, where the prevalence of MDR-TB is increasing.

## Abbreviations

AFB: Acid fast bacilli
DNA: Deoxyribonucleic acid
DST: Drug susceptibility testing
GENETUP: German-Nepal Tuberculosis Project
INH: Isoniazid
MDR-TB: Multidrug resistant tuberculosis
MTB: *Mycobacterium tuberculosis*
NHRC: Nepal Health Research Council
NPV: Negative predictive value
NTM: Nontuberculous Mycobacteria
PCR: Polymerase chain reaction
PPV: Positive predictive value
RIF: Rifampicin
TB: Tuberculosis
WHO: World Health Organization
